# Effect of remdesivir on adverse kidney outcomes in hospitalized patients with COVID-19 and impaired kidney function

**DOI:** 10.1371/journal.pone.0279765

**Published:** 2023-02-27

**Authors:** Rituvanthikaa Seethapathy, Qiyu Wang, Sophia Zhao, Ian A. Strohbehn, Joshua D. Long, James E. Dinulos, Destiny Harden, Vinay B. Kadiyala, Daiana Moreno, Meghan E. Sise

**Affiliations:** 1 Division of Nephrology, Department of Medicine, Massachusetts General Hospital, Boston, MA, United States of America; 2 Department of Medicine, Mass General Brigham Salem Hospital, Salem, MA, United States of America; Hospital Femina, BRAZIL

## Abstract

**Background:**

Chronic kidney disease (CKD) is an important risk factor for mortality from COVID-19. Remdesivir has been shown to shorten time to recovery in patients with severe COVID-19. However, exclusion of patients with severe kidney function impairment in clinical trials has led to concerns about kidney safety of remdesivir in patients with pre-existing kidney disease.

**Methods:**

Retrospective propensity score matched cohort study of hospitalized patients with COVID-19 admitted with estimated glomerular filtration rate (eGFR) between 15 − 60 mL/min/1.73m^2^. Remdesivir-treated patients were 1:1 matched to historical comparators admitted during the first wave of COVID-19 (between March-April 2020) prior to emergency use authorization of remdesivir using propensity scores accounting for factors predicting treatment assignment. Dependent outcomes included in-hospital peak creatinine, incidence of doubling of creatine, rate of kidney replacement therapy initiation and eGFR among surviving patients at day 90.

**Results:**

175 remdesivir-treated patients were 1:1 matched to untreated historical comparators. Mean age was 74.1 (SD 12.8), 56.9% were male, 59% patients were white, and the majority (83.1%) had at least one co-morbidity. There were no statistically significant differences in peak creatinine during hospitalization (2.3mg/dL vs. 2.5 mg/dL, *P* = 0.34), incidence of doubling of creatinine (10.3% vs. 13.1%, *P* = 0.48), and rate of kidney replacement therapy initiation (4.6% vs. 6.3%, *P* = 0.49) in remdesivir-treated patients versus matched untreated historical comparators, respectively. Among surviving patients, there was no difference of the average eGFR at day 90 (54.7 ± 20.0 mL/min/1.73m^2^ for remdesivir-treated patients vs. 51.7 ± 19.5 mL/min/1.73m^2^ for untreated comparators, *P* = 0.41).

**Conclusions:**

Remdesivir use in patients with impaired kidney function (eGFR between 15 − 60 mL/min/1.73m^2^) who present to the hospital with COVID-19 is not associated with increased risk of adverse kidney outcomes.

## Introduction

Remdesivir was the first antiviral therapy approved by the Food and Drug Administration for treatment of coronavirus disease 2019 (COVID-19). It has been shown to shorten time to recovery in hospitalized adults with pneumonia [1], and a recent randomized clinical trial also demonstrated its efficacy in the outpatient setting to prevent hospitalization [2]. Chronic kidney disease (CKD) has been recognized as a key risk factor for severe COVID-19 and mortality [3–5]. However, patients with estimated glomerular filtration rate (eGFR) < 30mL/min/1.73m^2^ were excluded from registrational trials of remdesivir, and there has been ongoing concern that remdesivir may be nephrotoxic, especially in patients with pre-existing CKD. Initially developed as an antiviral therapy during the 2014 Ebola outbreak, remdesivir has limited water solubility and requires a sulfobutylether-beta-cyclodextrin (SBECD) excipient for intravenous delivery. SBECD is a large oligosaccharide that is eliminated by glomerular filtration. In animal studies, high doses (approximately 50-fold greater than the dose administered in clinical use) can lead to renal tubular vacuolization and foamy macrophages in the liver [6]. For hospitalized patients, remdesivir is typically prescribed for five doses (200mg intravenous loading dose on day 1, followed by 100mg daily on days 2 through 5). Real world data regarding nephrotoxicity of remdesivir remains controversial: multiple case series have suggested that remdesivir may be safe in patients with advanced kidney disease [7–12], however, analysis of pharmacovigilance databases demonstrated higher reports of kidney injury associated with remdesivir compared to other medications used to treat COVID-19 [13, 14].

Thus, there remains clinical equipoise on whether remdesivir should be used in patient with kidney function impairment who present with moderate to severe COVID-19. To provide further evidence on kidney safety, we designed a propensity-score matched study to compare four adverse kidney outcomes (peak creatinine value, rate of doubling of creatinine, rate of kidney replacement therapy initiation, and eGFR at day 90) among hospitalized patients with COVID-19 who were treated with remdesivir and historical comparators who did not receive remdesivir.

## Methods

### Patients and propensity score matching

**Fig 1:**
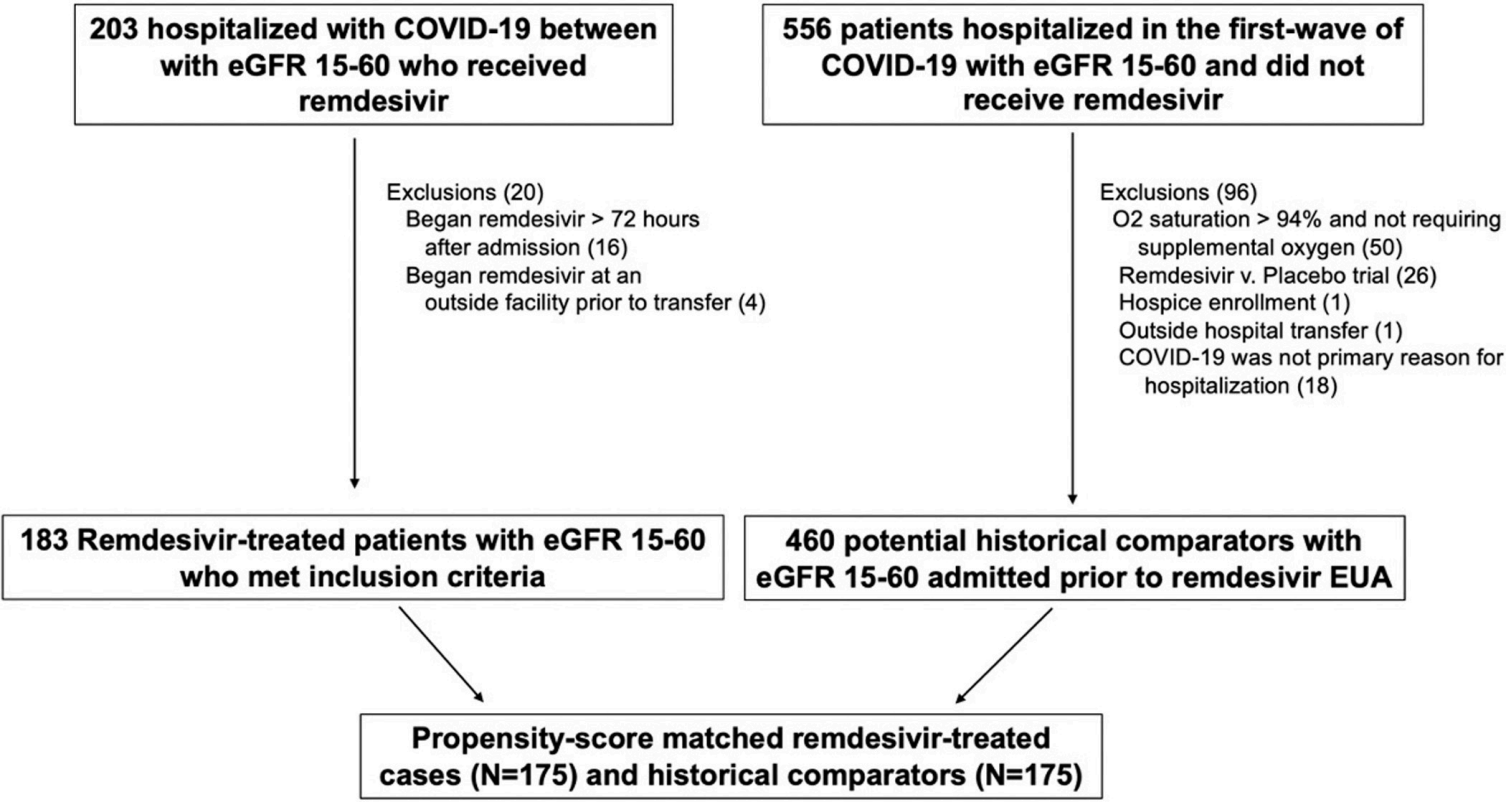
Patient flow. Historical comparators were obtained from the first wave of COVID-19 in Boston, Massachusetts and had been admitted between March 17, 2020 and April 30, 2020. Remdesivir treated patients were admitted between April 21, 2020 and November 29, 2020. Abbreviations: eGFR = estimated glomerular filtration rate, EUA = emergency use authorization.

We included adult patients (≥18 years old) who were admitted to Mass General Brigham (MGB) healthcare system with COVID-19 whose admission creatinine corresponded to an eGFR value between 15 − 60mL/min/1.73m^2^ [15]. Remdesivir-treated patients received at least one dose of remdesivir, and had at least one repeat creatinine measurement after receiving remdesivir. Remdesivir-treated patients were excluded if they received remdesivir >72 hours after admission, or if they received remdesivir at an outside hospital prior to being transferred to our healthcare network. Historical comparators were adult patients admitted to MGB during the first wave of COVID-19 in Boston, MA between March-April 2020 (prior to the emergency use authorization of remdesivir) and had an admission creatinine that corresponded to an eGFR value between 15 − 60mL/min/1.73m^2^. Comparators were excluded if they had end-stage kidney disease (ESKD) or if they were placed into hospice/comfort care prior to receiving therapy for COVID-19 (**Fig 1**).

In order to control for confounding factors that are associated with both treatment assignment and outcome [16], we performed propensity score matching between the remdesivir-treated cohort and the untreated historical comparators. Patients were matched on independent variables associated with the administration of remdesivir, including age, sex, race/ethnicity, diabetes, hypertension, CKD (defined by eGFR < 60mL/min/1.73m^2^ sustained for at least 90 days), kidney transplantation, admission creatinine, and markers of disease severity on hospital admission, including need for mechanical ventilation and the highest sequential organ failure assessment (SOFA) score within the first 12 hours of admission [17]. We used logistic regression to estimate the propensity of receiving remdesivir, and performed 1:1 nearest-neighbor matching without replacement with a caliper of 0.1 standard deviation of the propensity score [18]. Standardized mean difference was calculated to evaluate the quality of the match [16].

### Study outcomes

The primary dependent outcome was the peak creatinine level during hospitalization in remdesivir-treated patients compared to untreated historical comparators. The secondary dependent outcomes were 1) incidence of doubling of creatinine from admission; 2) initiation of kidney replacement therapy (either continuous or intermittent dialysis modality); and the 3) average eGFR among patients who were alive at day 90. To determine the eGFR at day 90, we evaluated all creatinine values between 75 to 180 days post-hospital admission and selected the value closest to day 90.

### Statistical analysis

Paired t-test or Wilcoxon signed-rank test was used to compare peak creatinine, hospital length of stay, creatinine measurements between remdesivir-treated patients and their matched untreated historical comparators, as appropriate. Among patients who initiated kidney replacement therapy, a peak creatinine of 10mg/dL was imputed on the day of kidney replacement therapy initiation if pre-dialysis creatinine level was less than 10mg/dL. McNemar test was used to compare the incidence of doubling of creatinine from admission and rate of kidney replacement therapy initiation between remdesivir-treated patients and matched untreated historical comparators. Among surviving patients, we compared the average eGFR at day 90 by remdesivir treatment status using independent sample t-test, as not all matched pairs had available day 90 creatinine levels.

To evaluate the robustness of our findings we performed the following sensitivity analyses: using a paired t-test, we compared the peak creatinine among remdesivir-treated patients who received full course of therapy (≥ 5 doses) and among remdesivir-treated patients who had ≥ 5 creatinine measurements after starting remdesivir. Finally, we stratified the analysis of peak creatinine by admission eGFR (eGFR 15–29 mL/min/1.73m^2^ and 30–60 mL/min/1.73m^2^).

## Results

There were 203 individuals with admission eGFR between 15 − 60 mL/min/1.73m^2^ who received remdesivir; 16 patients were excluded due to initiating remdesivir more than 72 hours after hospital admission and 4 patients were excluded due to having received remdesivir at an outside hospital prior to transfer to our healthcare system, leaving 183 remdesivir-treated patients included. There were 556 potential historical comparators; after applying the exclusion criteria, we included 460 potential historical comparators hospitalized with COVID-19 between March and April 2020 in propensity score matching (**Fig 1**).

Prior to matching, historical comparators were older and had higher SOFA scores, reflecting the high acuity of COVID-19 in patients hospitalized in the first wave in Boston, Massachusetts. A sufficiently close match was found for 175 of the 184 (95%) remdesivir-treated patients. All patient characteristics achieved good balance (standardized mean differences < 0.1) (**Table 1**).

**Table 1:** Patient characteristics pre- and post-propensity score matching.

	Before propensity score matching				After propensity score matching			
***Characteristic***	Remdesivir-treated N = 183	Untreated historical comparators N = 460	P value	SD*	Remdesivir-treated N = 175	Untreated historical comparators N = 175	P value	SD*
Age (mean (SD))	73.2 (12)	76.9 (13)	0.001	-0.31	73.7 (12)	74.5 (14)	0.534	-0.06
Female (%)	76 (42)	234 (51)	0.040	0.09	75 (43)	76 (43)	1.000	0.006
White Race (%)	105 (57)	279 (61)	0.500	-0.03	101 (58)	107 (61)	0.576	-0.03
***Comorbidities***								
Hypertension (%)	147 (80)	364 (79)	0.812	0.01	140 (80)	144 (82)	0.677	-0.02
Diabetes (%)	108 (59)	216 (47)	0.008	0.12	100 (57)	100 (57)	1.000	0.00
Preexisting CKD (%)	86 (47)	207 (45)	0.71	0.02	82 (47)	73 (42)	0.380	0.05
Kidney transplant (%)	3 (1.6)	7 (1.5)	1.000	0.001	2 (1)	3 (2)	1.000	-0.0057
***Disease severity at presentation***								
Invasive ventilation (%)	20 (11)	78 (17)	0.072	-0.06	20 (11)	17 (10)	0.735	0.08
SOFA Score (mean (SD))	3.42 (2.5)	4.09 (3.0)	0.007	-0.27	3.50 (3)	3.31 (2)	0.459	0.07
Admission creatinine (mean (SD))**	1.61 (0.54)	1.62 (0.57)	0.79	-0.02	1.61 (0.5)	1.58 (0.6)	0.673	0.05

Each remdesivir-treated patient was matched 1:1 with a potential comparator who was admitted in March or April 2020 prior to the emergency use authorization of remdesivir. The results presented here are the means (standard deviations) or counts (percentage).

* SD (standardized [mean] difference) is a measure of distance between two group means in terms of a variables and is a conventional metric used to evaluate the quality of a match. By convention, a SD of less than 0.1 is considered a well-balanced covariate.

**There were 32 remdesivir-treated patients (18%) with presenting eGFR 15-29mL/min/1.73m^2^, 71 (41%) with eGFR 30-44mL/min/1.73m^2^, and 72 (41%) with eGFR 45-59mL/min/1.73m^2^. In the propensity score matched untreated historical comparators the breakdown was 33 (19%) with eGFR 15–29 mL/min/1.73m^2^, 60 (34%) 30-44mL/min/1.73m^2^, 82 (47%) 45-59mL/min/1.73m^2^. Abbreviations: SD = standard deviation, CKD = chronic kidney disease, SOFA = sequential organ failure assessment.

In the matched cohort, mean age was 74 (SD 13), 56.9% were male, and 59.0% patients were white. The majority of patients had at least one co-morbidity (83.1%), and 44.3% had pre-existing CKD. A total of 10.6% received mechanical ventilation within 12 hours of presentation. There were no significant differences in hospital length of stay or total number of creatinine measurements between the groups: median length of stay was 8 days (IQR 6–17) and number of creatinine measurements was 11 (IQR 7–21) in remdesivir-treated patients; while median length of stay was 9 days (IQR 5–16), and number of creatinine measurements was 10 (IQR 6–23) in untreated historical comparators (Wilcoxon signed-rank test, *P* = 0.38 and *P* = 0.1, respectively). The majority of patients (141 patients, [81%]) received a full course of remdesivir (≥ 5 days). Among the remaining 34 patients who received shorter course of remdesivir (<5 doses), the reasons for early discontinuation included: rapid recovery (19), worsening kidney function (4), elevated transaminases (2), anaphylaxis (1), transition to comfort care (6) and uncertain causes (2).

### Peak creatinine level during hospitalization

**Fig 2:**
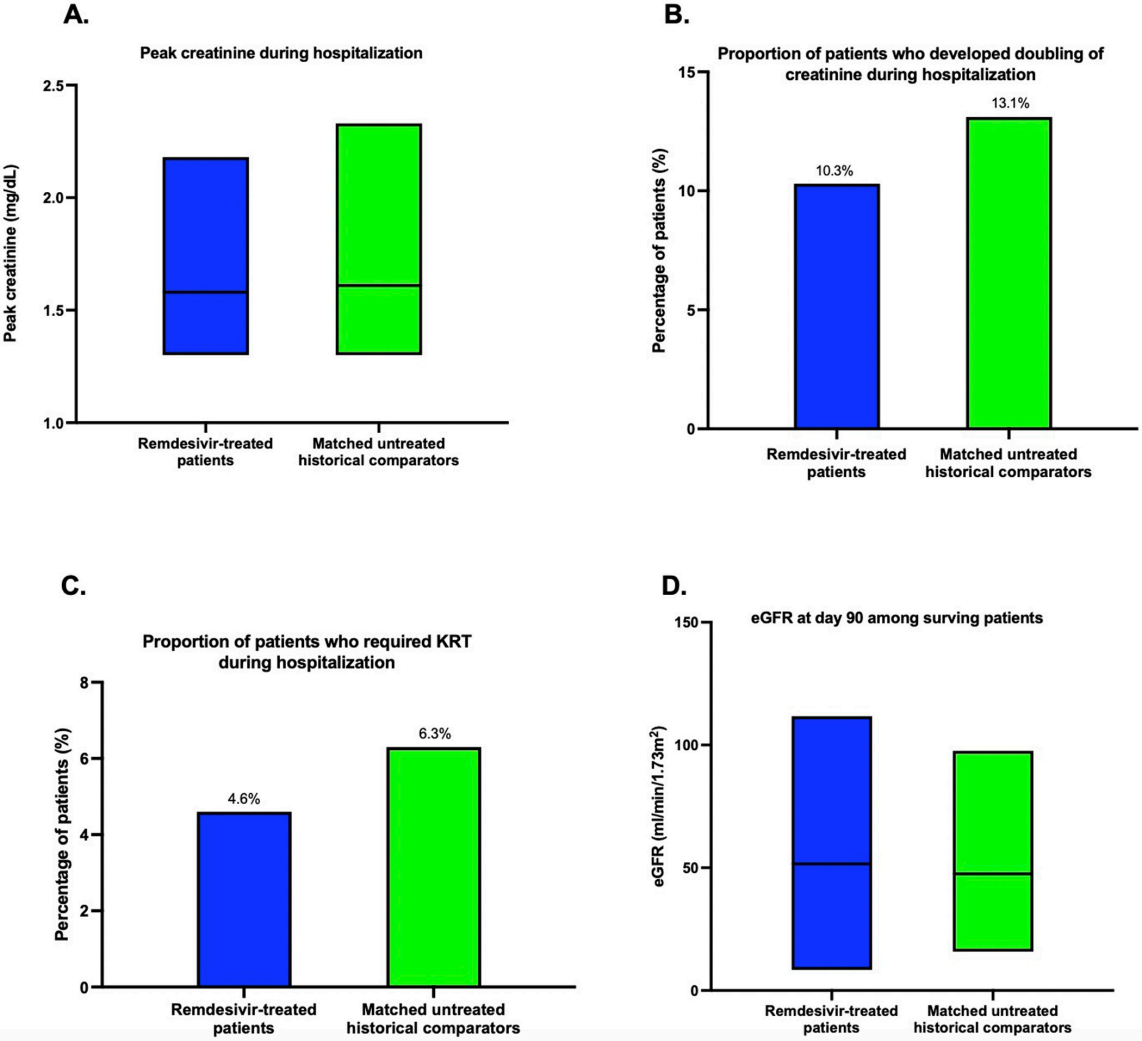
Kidney outcomes in remdesivir-treated patients and propensity score matched untreated historical comparators. There was no statistically significant difference in any of the adverse kidney outcomes. A) Peak creatinine during hospitalization B) Percentage of patients developed doubling of creatinine C) Percentage of patients requiring initiation of kidney replacement therapy D) eGFR at day 90 among surviving patients. Boxplot (2A, 2D) showed the 1^st^ quartile, median and 3^rd^ quartile of peak creatinine and day 90 eGFR, respectively. Abbreviation: KRT: kidney replacement therapy; eGFR: estimated glomerular filtration rate.

Mean peak creatinine in the remdesivir-treated group was 2.3 mg/dL (95% confidence interval [CI] 1.98–2.57) compared to 2.5 mg/dL (95% CI 2.13–2.89) in matched untreated historical comparators (Paired t test, *P* = 0.34) (**Fig 2A**). Sensitivity analyses including only the subset of patients who received full course of remdesivir and those with at least 5 post-treatment creatinine measurements and their matched historical comparators did not identify differences in peak creatinine (**S1A and S1B Fig**). Stratification of remdesivir-treated patients by their admission eGFR also did not affect the comparison of peak creatinine during hospitalization (**S2A and S2B Fig**).

### Incidence of doubling of creatinine

Eighteen of the remdesivir-treated patients (10.3%) versus 23 of the matched untreated historical comparators (13.1%) experienced a doubling of serum creatinine during their hospitalization (McNemar test, *P* = 0.48) (**Fig 2B**).

### Incidence of renal replacement therapy initiation

Eight (4.6%) of the remdesivir-treated patients and 11 (6.3%) of the matched untreated historical comparators received kidney replacement therapy during hospitalization, respectively (McNemar test, *P* = 0.49) (**Fig 2C**).

### Day 90 eGFR among surviving patients

Among surviving patients to who were followed for at least 90 days post admission (N = 120), the average eGFR at day 90 was 54.7 mL/min/1.73m^2^ (SD 20.0) in remdesivir-treated patients (N = 66) compared to 51.7 mL/min/1.73m^2^ (SD 19.5) among untreated historical comparators (N = 54) (Independent sample t test, *P* = 0.41) (**Fig 2D**).

## Discussion

In this propensity score matched retrospective cohort study, we did not detect significant differences in adverse kidney outcomes including peak serum creatinine, rate of doubling of serum creatinine, need for kidney replacement therapy, and eGFR at day 90 between patients who received remdesivir and those who did not. Our findings provide additional safety signal and are complementary to previous studies suggesting that off-label remdesivir use in patients with ESKD and eGFR < 30mL/min/1.73m^2^ is safe and well tolerated [7–12]. Prior studies that identified a signal for remdesivir nephrotoxicity used pharmacovigilance databases, which may be limited by reporting bias making it challenging to generalize causal association [13, 14]. Thus, it is important to include untreated comparators, particularly given the high baseline rate of acute kidney injury among patients hospitalized for COVID-19 (ranging from 17~56%) [19–23].

Exclusion and under-representation of patients with kidney disease in clinical trials of life-saving treatments is a major problem that has been magnified by the COVID-19 pandemic [24–26]. Studies have noted that kidney disease was an exclusion criteria in approximately half of the registered trials evaluating therapeutics for COVID-19, including all of the major trials that led to the approval of the antiviral agents for COVID-19 (remdesivir, nirmaltrelvir/ritonavir, and molnupiravir) [1, 25–28]. Thus, there is an urgent need to address this knowledge gap using real-world data, as patients with kidney disease suffer significantly higher morbidity and mortality from COVID-19. Numerous studies have shown ESKD and advanced CKD are among the top comorbid medical conditions associated with death in patients with COVID-19 [3, 5, 29, 30]. Recent studies show that even after vaccination, the risk of hospitalization and death is substantial in patients with ESKD who develop COVID-19; up to 45% of patients with breakthrough infection may be hospitalized [31], and up to 7% may die within 28 days [32]. This highlights the importance of including this particularly vulnerable population into clinical trials for evaluation of antiviral and immunomodulating treatments for COVID-19.

Our study has several limitations: First, we chose historical controls from the first wave of COVID-19 prior to emergency use authorization of remdesivir to avoid confounding by indication and minimize selection bias. We performed detailed chart review to ensure accurate information on disease severity and other baseline covariates that are associated with the likelihood of remdesivir administration and achieved good balance between the remdesivir-treated patients and matched untreated comparators after propensity score matching. However, the rapidly changing standard of care could have impacted the disease outcome and kidney injury rate [20], which could not be fully adjusted for by propensity score matching. For example, the effect of dexamethasone use could not be controlled for as all historical comparators were admitted prior to routine use of dexamethasone at our center [33]. Second, our outcome of eGFR at day 90 was limited by the fact that many surviving patients did not have creatinine values within our healthcare system available within the 75 to 180 days window, raising concern for informative censoring. Fortunately, loss to follow-up was similar in each group. Third, creatinine-based GFR estimating equations have low sensitivity for detecting renal dysfunction in critically ill patients who have low creatinine production, thus the rate of acute kidney injury and more importantly, the development of CKD in survivors may be underestimated [34].

## Conclusions

Our report adds to a growing body of data suggesting that in patients presenting to the hospital with impaired kidney function (eGFR 15–60mL/min/1.73m^2^), remdesivir is not associated with short-term adverse kidney outcomes.

## Supporting information

S1 Fig Sensitivity analyses that included only remdesivir-treated patients who had a least 5 creatinine measurements after treatment initiation (A) and those who received full course (≥5 doses) of remdesivir (B) showing no statistically significant difference in peak creatinine compared to their matched untreated historical comparators (paired t test, P = 0.70 and P = 0.57, respectively). Boxplot showing the 1st quartile, median and 3rd quartile of peak creatinine distribution; numbers above the box represent mean and standard deviation.
(PDF)

S2 Fig Stratification by admission eGFR showed no significant differences between peak creatinine among those with admission eGFR between 30-60mL/min/1.73m2 (A) and those with admission eGFR between 15–30 mL/min/1.73m2 (B) (independent t test, P = 0.23 and P = 0.75, respectively). Analyses were not performed among matched pairs as patients were not matched based on exact admission eGFR and were instead matched based on propensity score (see Method section, patients and propensity score matching). Boxplot showing the 1st quartile, median and 3rd quartile of peak creatinine; numbers above the box represent mean and standard deviation.
(PDF)

S1 Dataset (XLS)
