# Supplementary material for: Effect of remdesivir on adverse kidney outcomes in hospitalized patients with COVID-19 and impaired kidney function
Source: PLoS One. 2023 Feb 27;18(2):e0279765. doi: 10.1371/journal.pone.0279765 (PMC9970064; doi:10.1371/journal.pone.0279765)
Supplement: S1 Fig — Sensitivity analyses that included only remdesivir-treated patients who had a least 5 creatinine measurements after treatment initiation (A) and those who received full course (≥5 doses) of remdesivir (B) showing no statistically significant difference in peak creatinine compared to their matched untreated historical comparators (paired t test, P = 0.70 and P = 0.57, respectively). Boxplot showing the 1st quartile, median and 3rd quartile of peak creatinine distribution; numbers above the box represent mean and standard deviation. (PDF) [file pone.0279765.s001.pdf]

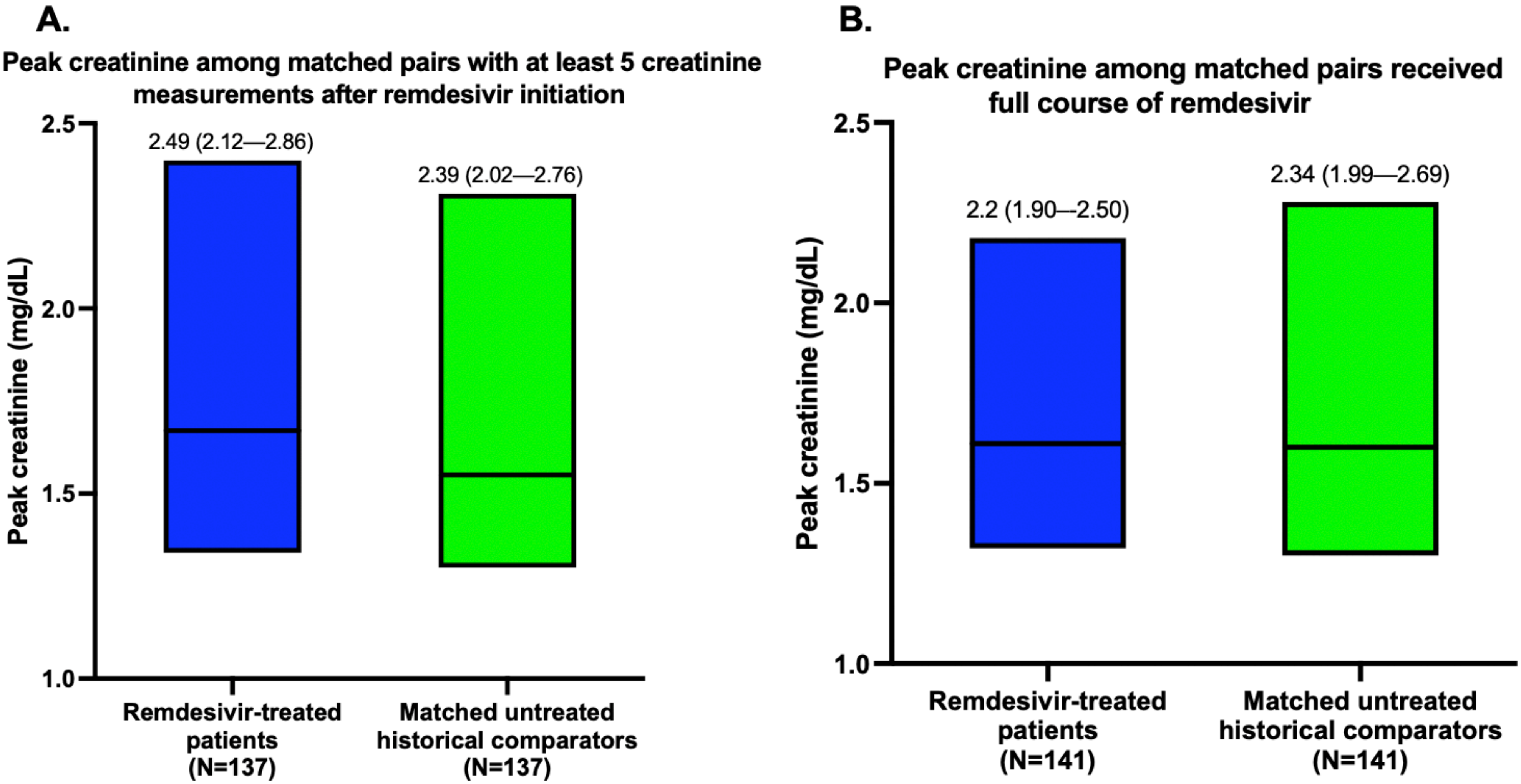
A.
Peak creatinine among matched pairs with at least 5 creatinine measurements after remdesivir initiation
2.5
2.0
1.5
1.0
Peak creatinine (mg/dL)
2.49 (2.12—2.86)
2.39 (2.02—2.76)
Remdesivir-treated patients (N=137)
Matched untreated historical comparators (N=137)
B.
Peak creatinine among matched pairs received full course of remdesivir
2.5
2.0
1.5
1.0
Peak creatinine (mg/dL)
2.2 (1.90—2.50)
2.34 (1.99—2.69)
Remdesivir-treated patients (N=141)
Matched untreated historical comparators (N=141)
