# Supplementary material for: Effect of remdesivir on adverse kidney outcomes in hospitalized patients with COVID-19 and impaired kidney function
Source: PLoS One. 2023 Feb 27;18(2):e0279765. doi: 10.1371/journal.pone.0279765 (PMC9970064; doi:10.1371/journal.pone.0279765)
Supplement: S2 Fig — Stratification by admission eGFR showed no significant differences between peak creatinine among those with admission eGFR between 30-60mL/min/1.73m2 (A) and those with admission eGFR between 15–30 mL/min/1.73m2 (B) (independent t test, P = 0.23 and P = 0.75, respectively). Analyses were not performed among matched pairs as patients were not matched based on exact admission eGFR and were instead matched based on propensity score (see Method section, patients and propensity score matching). Boxplot showing the 1st quartile, median and 3rd quartile of peak creatinine; numbers above the box represent mean and standard deviation. (PDF) [file pone.0279765.s002.pdf]

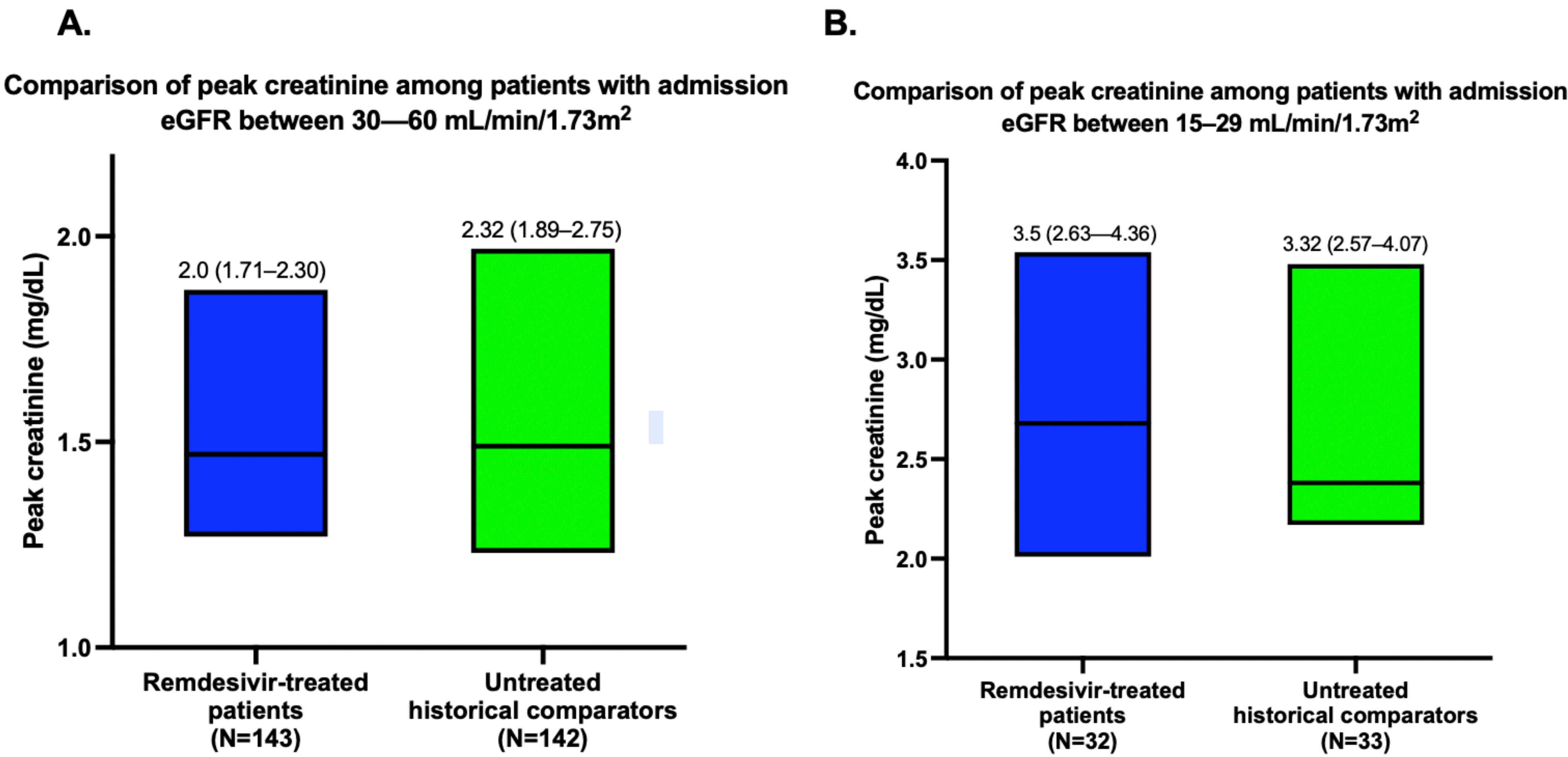
A.
Comparison of peak creatinine among patients with admission eGFR between 30—60 mL/min/1.73m2
Peak creatinine (mg/dL)
2.0
1.5
1.0
2.0 (1.71–2.30)
2.32 (1.89–2.75)
Remdesivir-treated patients (N=143)
Untreated historical comparators (N=142)
B.
Comparison of peak creatinine among patients with admission eGFR between 15–29 mL/min/1.73m2
Peak creatinine (mg/dL)
4.0
3.5
3.0
2.5
2.0
1.5
3.5 (2.63—4.36)
3.32 (2.57–4.07)
Remdesivir-treated patients (N=32)
Untreated historical comparators (N=33)
